# Microbiota-derived metabolite signatures in Alzheimer’s disease across the cognitive aging continuum: a human-focused systematic review and evidence-weighted knowledge graph

**DOI:** 10.3389/fmicb.2026.1925387

**Published:** 2026-09-28

**Authors:** Xiaoteng He, Lili Zhang, Caiqiu Song, Yulin Liu

**Affiliations:** 1 Jiangxi University of Chinese Medicine, Nanchang, Jiangxi, China; 2 The Second Affiliated Hospital of Nanchang University, Nanchang, Jiangxi, China

**Keywords:** Alzheimer’s disease, bile acids, cognitive aging, gut microbiota, knowledge graph, metabolomics, microbiota-derived metabolites, short-chain fatty acids

## Abstract

**Background:**

Gut microbial metabolism is increasingly implicated in Alzheimer’s disease (AD), mild cognitive impairment (MCI), preclinical amyloid accumulation, and cognitive aging. However, existing reviews often focus on microbial taxonomy rather than the metabolite mediators that connect the gut ecosystem with AD pathology, neuroinflammation, and cognition.

**Objectives:**

To systematically synthesize human and translational evidence on microbiota-derived or microbiota-associated metabolite signatures across the cognitive aging continuum using structured evidence synthesis, evidence mapping, and an evidence-weighted knowledge graph.

**Methods:**

A systematic search of PubMed, the Web of Science Core Collection, and Academic Search Premier via EBSCOhost was conducted to identify eligible studies. Citation checking and targeted full-text retrieval were used during screening and evidence verification. After de-duplication, records were screened by population, exposure or intervention, comparator, outcome layer, and study design. Human core, human bridge, human intervention, mechanistic/translational, and resource/background-mechanism evidence were coded separately. Module-level quantitative summaries were retained only where data were sufficiently comparable; otherwise, structured direction-of-effect synthesis and evidence mapping were used. Overlapping Alzheimer’s Disease Neuroimaging Initiative (ADNI) bile-acid analyses were synthesized as complementary outcome layers rather than treated as independent replications of the same association.

**Results:**

The final evidence pool contains 60 records, including 32 direct, bridge, or intervention human evidence records and 28 mechanistic, translational, animal, cellular, resource, or background-mechanism records. Bile-acid profiles and short-chain fatty acid (SCFA)-related functional capacity were recurrent human evidence modules. Trimethylamine N-oxide (TMAO)/choline-related findings were context dependent, varying by biological matrix, population, renal function, and cardiometabolic context, whereas selected tryptophan/indole/kynurenine pathways remained emerging. Across these metabolite modules, the evidence-weighted knowledge graph showed recurrent convergence on barrier dysfunction, inflammatory and glial signaling, and mitochondrial stress, while retaining context-dependent conflicts and gaps in human validation.

**Conclusion:**

The current literature points to a convergent network of microbiota-related metabolic pathways rather than a single metabolite marker or causal route. The synthesis prioritizes external validation of bile-acid signatures, matched multi-compartment assessment of SCFA- and TMAO-related pathways, and direct human testing of indole/kynurenine and lipid-like mechanisms. These priorities require longitudinal and interventional studies with harmonized metabolomics, AD biomarkers, and cognitive outcomes.

## Introduction

Alzheimer’s disease (AD) develops across a long cognitive-aging continuum that extends from apparently normal cognition and preclinical amyloid accumulation to subjective cognitive decline, mild cognitive impairment (MCI), and dementia. Although amyloid and tau remain central defining features of AD biology, clinical heterogeneity, inflammatory comorbidity, metabolic vulnerability, and variable intervention responsiveness suggest that AD cannot be explained by brain pathology alone. In this context, the gut microbiota has emerged as a modifiable system that may influence host metabolism, immune tone, intestinal barrier integrity, and brain function (Kivipelto et al., 2006; Bateman et al., 2012; Villemagne et al., 2013; Livingston et al., 2020; Livingston et al., 2024; Jimenez-García et al., 2024; Jemimah et al., 2023; Li et al., 2024).

Much of the early AD microbiome literature focused on bacterial composition, reporting shifts in taxa such as Bacteroides, Firmicutes, Proteobacteria, Akkermansia, Faecalibacterium, Blautia, Agathobacter, Ruminococcus, and other SCFA-producing or proinflammatory organisms. Taxonomic signatures are important but inconsistent across cohorts and platforms. Prior systematic and scoping reviews likewise found limited reproducibility of recurring taxa and substantial geographic and methodological concentration, supporting a shift toward functional metabolite-level synthesis (Jemimah et al., 2023; Warren et al., 2026). Taxa alone rarely explain how gut dysbiosis becomes biologically meaningful for amyloid deposition, tau phosphorylation, synaptic vulnerability, neuroinflammation, or cognitive decline. Metabolites provide a more direct functional layer because they represent chemical outputs of host–microbe interactions and can act on epithelial, immune, vascular, endocrine, and neural pathways (Cryan et al., 2019; Cryan et al., 2020; Morais et al., 2021; Yin et al., 2025; Vogt et al., 2017; Cattaneo et al., 2017; Harach et al., 2017; Minter et al., 2016; Zhuang et al., 2018; Liu et al., 2019).

Microbiota-derived and microbiota-associated metabolites span several biologically distinct classes. Short-chain fatty acids (SCFAs), especially acetate, propionate, and butyrate, are fermentation products that influence intestinal barrier function, immune regulation, microglia, mitochondrial homeostasis, and energy metabolism. Bile acids reflect host-microbial co-metabolism and have been associated with AD diagnosis, cognitive outcomes, cerebrospinal fluid biomarkers, amyloid and tau PET, and neuroimaging markers. Trimethylamine N-oxide (TMAO), derived from microbial processing of choline-related substrates followed by host hepatic oxidation, has been linked to tau, neurodegeneration, oxidative stress, synaptic dysfunction, and cognition. Tryptophan-derived indoles, kynurenine-pathway metabolites, and related immune-neurochemical signals may influence aryl hydrocarbon receptor (AhR), pregnane X receptor (PXR), inflammation, amyloid burden, and neuronal resilience. Emerging lipid-like metabolites, including lysophosphatidylcholine, and volatile organic compounds add further mechanistic diversity (Dalile et al., 2019; Morrison and Preston, 2016; Wahlström et al., 2016; Vogt et al., 2018; Agus et al., 2018; Roager and Licht, 2018; Gheorghe et al., 2019; Ubeda et al., 2023; Zha et al., 2025).

The field has recently moved beyond cross-sectional associations. Human studies now include multi-biofluid metabolomics, fecal metagenomic functional pathway analysis, amyloid-stratified preclinical cohorts, paired plasma–fecal multiomics, and intervention trials involving ketogenic or Mediterranean ketogenic diets, multinutrient formulations, probiotics, acupuncture-associated multiomics shifts, and computerized cognitive training. In parallel, mechanistic studies have tested specific microbiota-metabolite-brain pathways such as *Bacteroides ovatus*–lysophosphatidylcholine–GPR119–ferroptosis, *Akkermansia muciniphila*–propionate–GPR41/GPR43–mitochondrial homeostasis, Agathobacter rectalis–butyrate–Akt/NF-κB–microglial signaling, and TMAO-related oxidative stress or tau mechanisms (Sonnenburg and Bäckhed, 2016; Nagpal et al., 2019; Schweickart et al., 2024; Zhao et al., 2025; Kuehn et al., 2025; Fu et al., 2026; Mo and Peng, 2026; Connell et al., 2026; Hosseinkhani et al., 2025; Molinero et al., 2025; Baek et al., 2025; Zhang et al., 2025; Wang et al., 2025; Dilmore et al., 2023; Dissanayaka et al., 2026).

Despite this rapid growth, synthesis remains difficult because studies differ in disease stage, sample matrix, analytical platform, metabolite annotation, outcome definition, cohort overlap, and effect reporting. This systematic review therefore used structured evidence synthesis, evidence mapping, module-level summaries where data were comparable, and direction-of-effect synthesis consistent with SWiM principles where pooling was inappropriate (Page et al., 2021; Campbell et al., 2020; Borenstein et al., 2021). Recent feasibility work integrating shotgun metagenomics, metrology, and multidimensional phenotyping across the cognitive-aging spectrum further illustrates both the promise and methodological demands of such designs (Frame et al., 2026).

Based on this rationale, this review focuses on the relationships between microbiota-derived or microbiota-associated metabolites and AD pathology, cognitive decline, and intervention responses across the cognitive aging continuum. Human evidence is treated as the core evidence base, while mechanistic and translational studies are used to interpret the biological context of these associations. This tiered synthesis helps identify metabolic modules that recur across studies, findings with relatively consistent human support, and pathways that warrant priority testing in future longitudinal cohorts and intervention trials (Figure 1).

**Figure 1:**
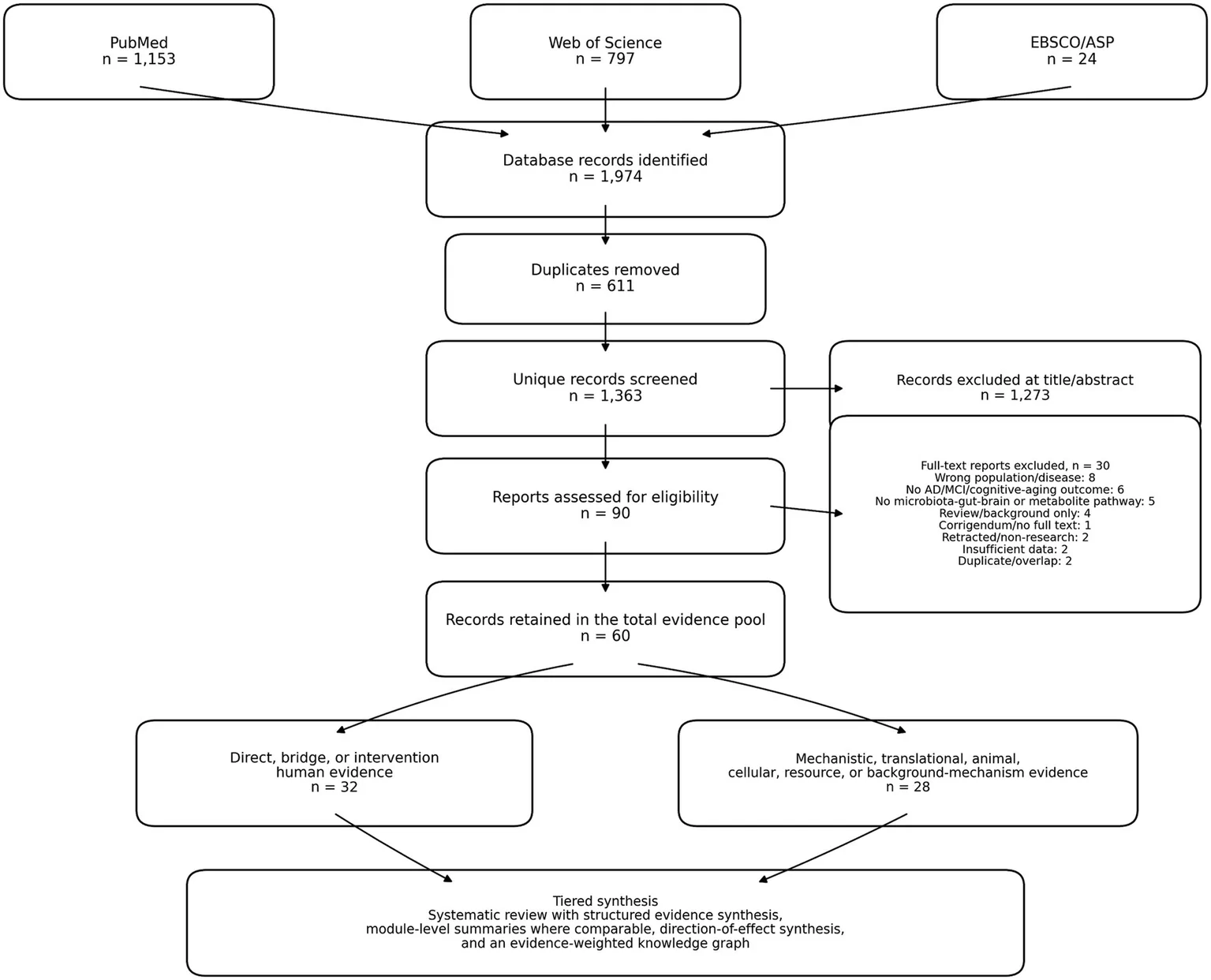
PRISMA screening and evidence assembly flow. The finalized flow reports 1,974 database records before deduplication, 611 duplicates removed, 1,363 unique records screened, 1,273 title/abstract exclusions, 90 full-text assessments, 30 full-text exclusions, and 60 retained evidence records. Supplementary Tables S1–S3 document the corresponding source counts, screening stages, and exclusion categories. The figure separates direct, bridge, or intervention human evidence from mechanistic, translational, animal, cellular, resource, or background-mechanism evidence.

## Methods

### Study design and review question

This systematic review examined whether microbiota-derived or microbiota-associated metabolites are associated with Alzheimer’s disease (AD) pathology, cognitive decline, or intervention response across the cognitive-aging continuum. Human evidence was the primary layer. Structured evidence synthesis, evidence mapping, and an evidence-weighted knowledge graph were used; module-level quantitative summaries were reserved for sufficiently comparable data. Core modules were SCFAs, bile acids, TMAO/choline-related metabolites, tryptophan/indole/kynurenine pathways, volatile or lipid-like metabolites, and multiomics signatures (Figure 2).

**Figure 2:**
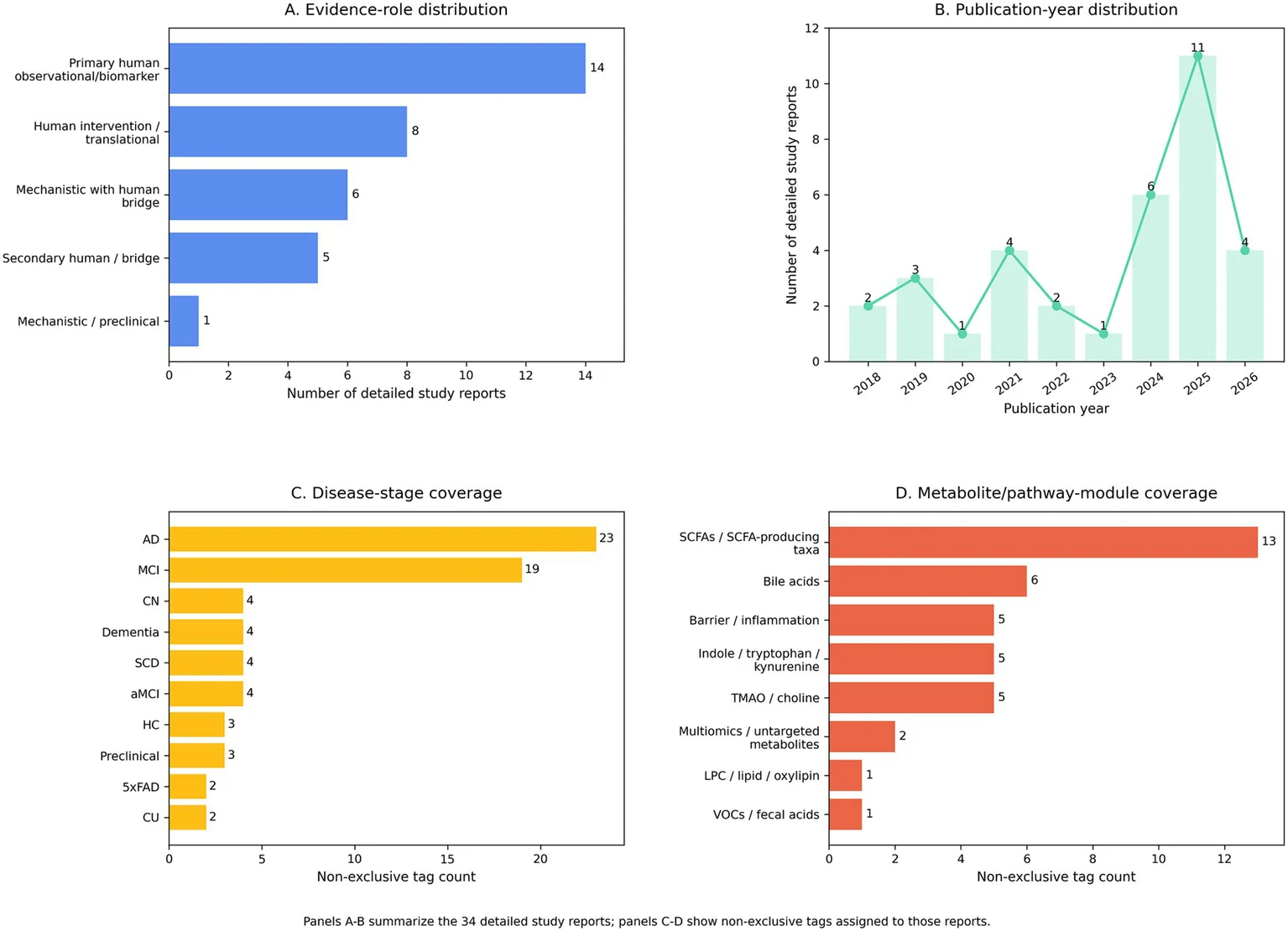
Evidence landscape across the Alzheimer’s disease and cognitive-aging continuum. The figure summarizes the 34 study-level reports detailed in Supplementary Table S4 and does not represent the complete 60-record evidence pool. Reports are organized by year, population stage, evidence role, metabolite module, and outcome layer; evidence density should not be interpreted as independent clinical replication. AD, Alzheimer’s disease; aMCI, amnestic mild cognitive impairment; CU, cognitively unimpaired; MCI, mild cognitive impairment; SCD, subjective cognitive decline.

The population, intervention or exposure, comparator, outcome, and study-design (PICOS) framework comprised adults with normal cognition, preclinical AD, subjective cognitive decline, mild cognitive impairment (MCI), or AD dementia; relevant translational models were included only as supporting evidence. Exposures or interventions were microbiota-related metabolites, metabolite-producing taxa, functional pathways, or targeted interventions. Comparators included control groups, lower-exposure strata, placebo/usual care, within-participant baselines, or biomarker-defined strata. Outcomes included cognition, AD biomarkers, amyloid/tau pathology, neuroimaging, neurodegeneration, inflammation, gut-barrier markers, and intervention-related change (McKhann et al., 2011; Sperling et al., 2011; Jessen et al., 2014; Jack et al., 2018; Petersen, 2016).

The review protocol was not prospectively registered. To limit *post hoc* methodological decisions, standardized metabolite modules, extraction fields, evidence-tier definitions, duplicate and cohort-overlap rules, direction codes, design-specific risk-of-bias tools, and graph-retention criteria were applied during extraction and synthesis (Figures 3–10).

**Figure 3:**
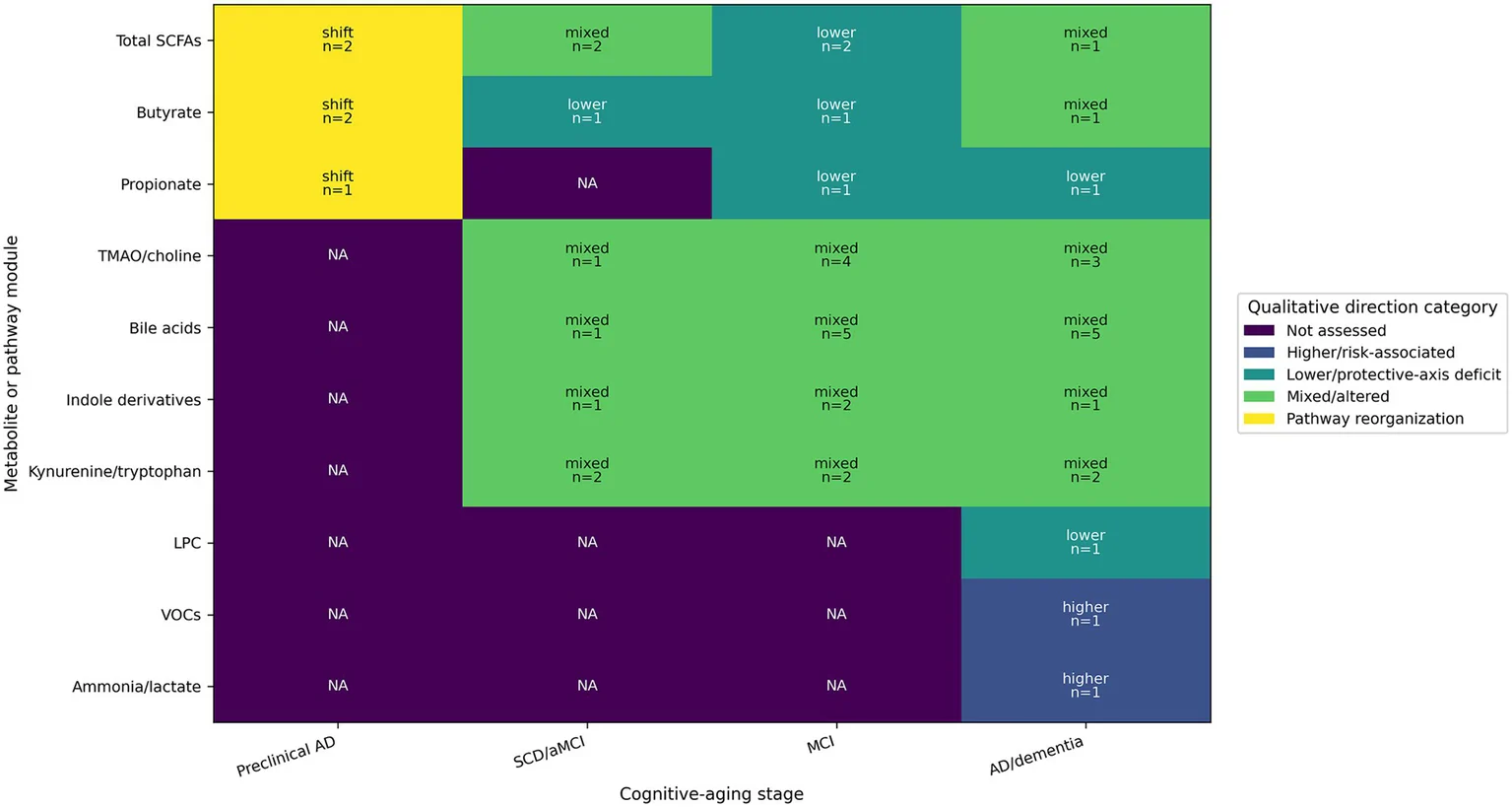
Direction-of-effect heatmap of microbiota-derived metabolite signatures. Rows represent metabolite modules and columns represent cognitive-aging stage. Cells display the number of contributing study reports; mixed-stage reports may contribute to more than one cell, and counts are not independent-cohort counts. Direction codes summarize increased, decreased, mixed, pathway-reorganized, intervention-responsive, or insufficient evidence rather than pooled causal effects. AD, Alzheimer’s disease; aMCI, amnestic mild cognitive impairment; MCI, mild cognitive impairment; SCD, subjective cognitive decline.

**Figure 4:**
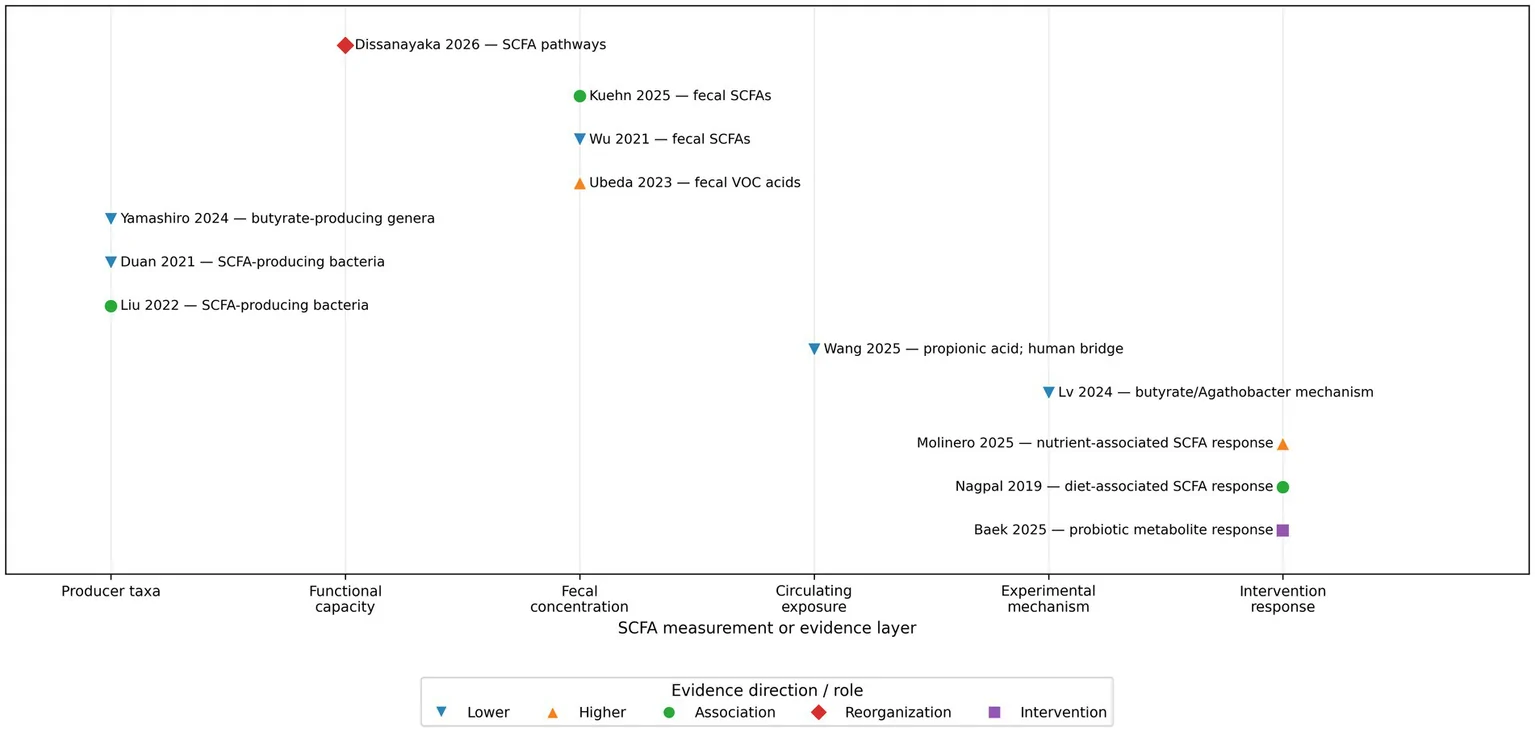
Short-chain fatty acid (SCFA) evidence module. SCFA-producing taxa, microbial production capacity, fecal concentration, circulating exposure, intervention findings, and mechanistic links are displayed as separate layers. The legend is positioned outside the data region so that all study labels remain visible. VOC, volatile organic compound.

**Figure 5:**
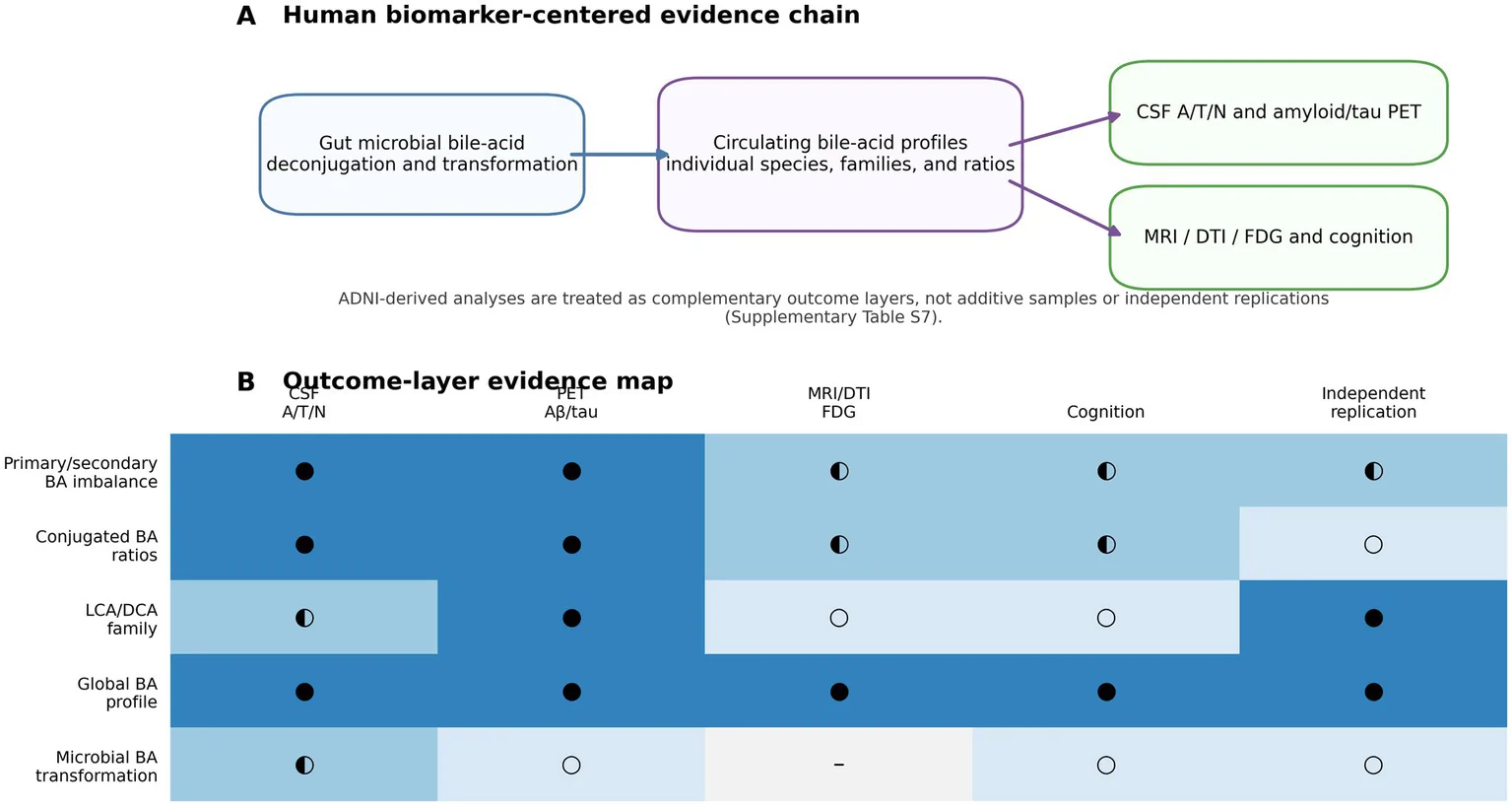
Bile-acid evidence across biomarkers and outcomes. **(A)** Human biomarker-centered evidence chain. **(B)** Outcome-layer evidence map. Symbols denote stronger or longitudinal support (●), repeated human association (◐), limited or indirect support (○), and not established (−). ADNI-derived analyses are treated as complementary layers rather than independent replications; detailed cohort and outcome mapping is provided in Supplementary Table S7. A/T/N, amyloid/tau/neurodegeneration; ADNI, Alzheimer’s Disease Neuroimaging Initiative; CSF, cerebrospinal fluid; DCA, deoxycholic acid; DTI, diffusion tensor imaging; FDG, fluorodeoxyglucose; LCA, lithocholic acid; MRI, magnetic resonance imaging; PET, positron emission tomography.

**Figure 6:**
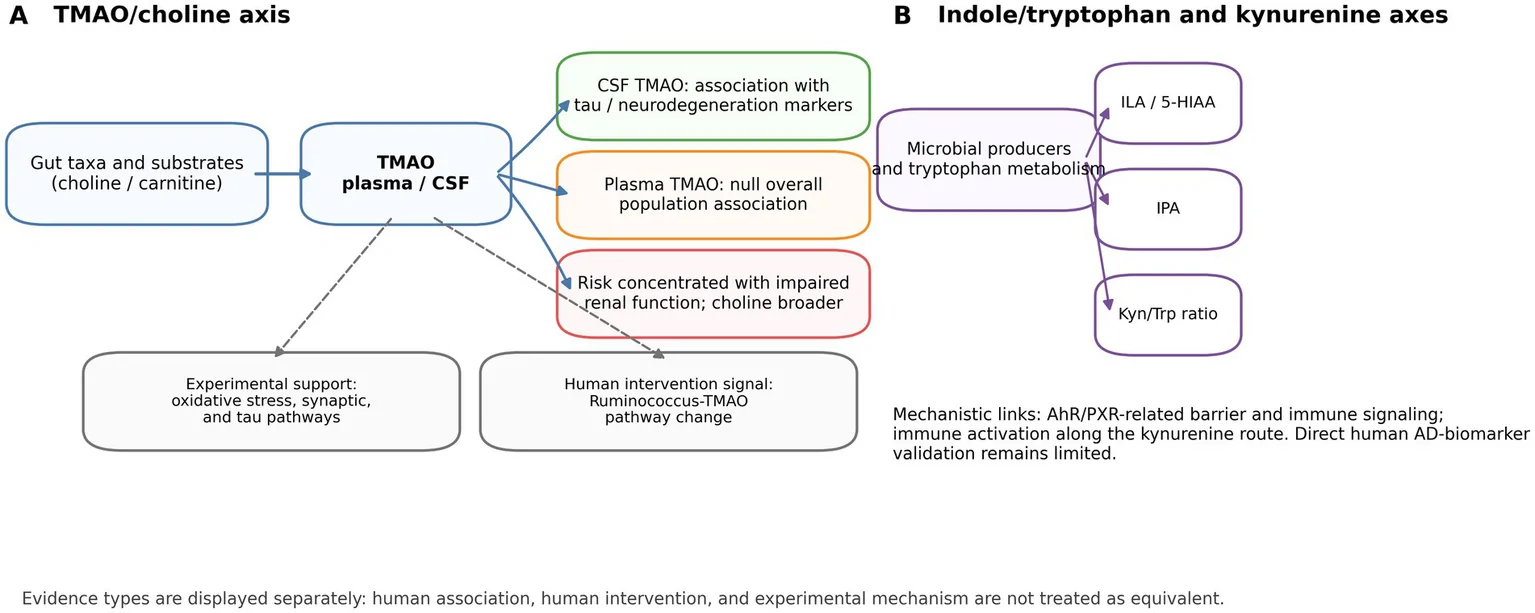
TMAO/choline and tryptophan-related evidence. **(A)** TMAO/choline axis. **(B)** Indole/tryptophan and kynurenine axes. Human associations, human intervention signals, and experimental mechanisms are displayed separately. The TMAO arm retains positive CSF findings, null overall plasma findings, and renal-function-dependent risk as distinct contexts; the indole and kynurenine arms emphasize mechanistic plausibility and limited direct human AD-biomarker validation. AhR, aryl hydrocarbon receptor; CSF, cerebrospinal fluid; 5-HIAA, 5-hydroxyindoleacetic acid; ILA, indole-3-lactic acid; IPA, indole-3-propionic acid; Kyn/Trp, kynurenine/tryptophan ratio; PXR, pregnane X receptor; TMAO, trimethylamine N-oxide.

**Figure 7:**
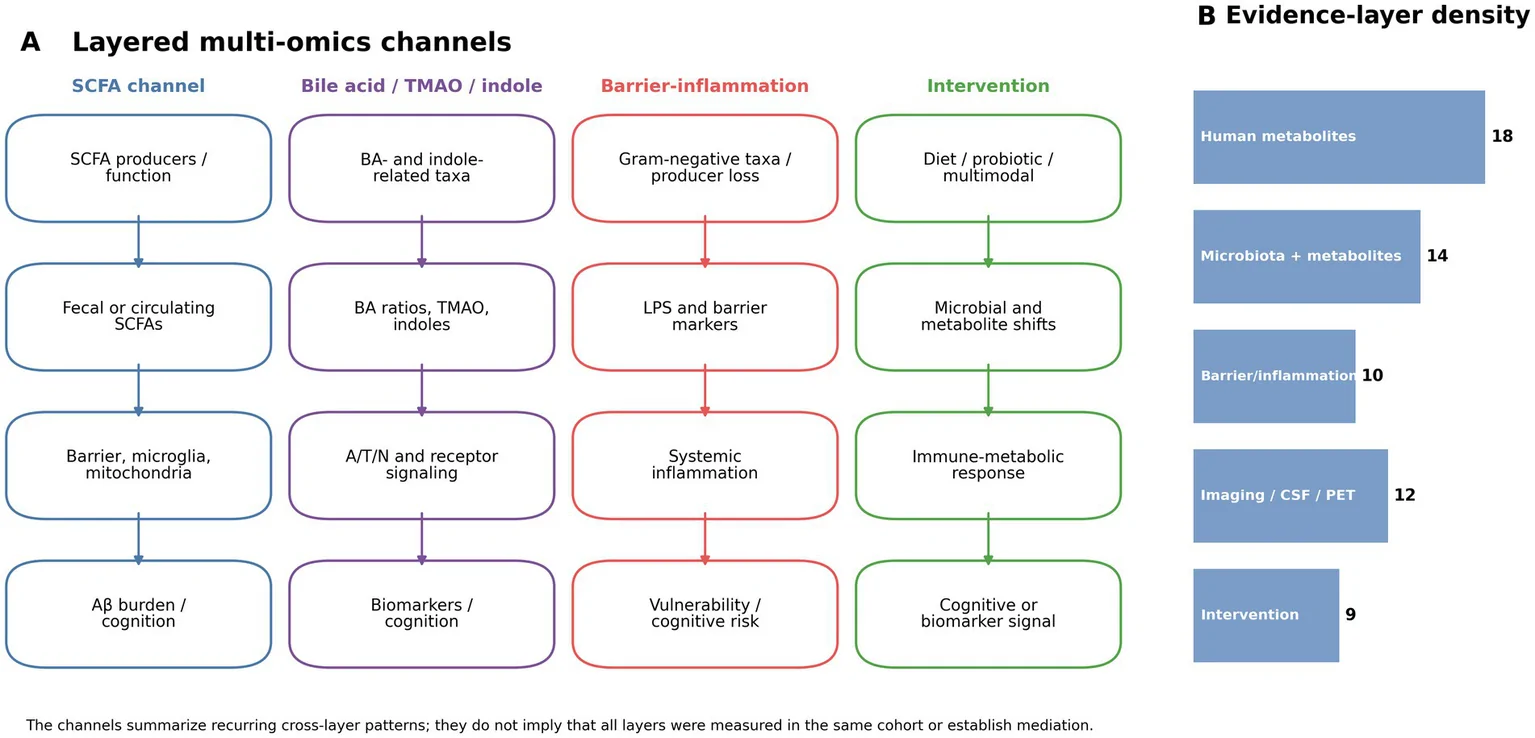
Multiomics and inflammation network. **(A)** Layered multi-omics channels showing gut microbial composition, fecal or circulating metabolites, host inflammatory or barrier markers, and brain/cognitive outcomes. **(B)** Evidence-layer density across the major evidence categories. Perioperative and stress-exposure studies are treated as bridge evidence, not direct AD causal evidence. Aβ, amyloid-beta; A/T/N, amyloid/tau/neurodegeneration; ADAS-Cog, Alzheimer’s Disease Assessment Scale-Cognitive Subscale; AhR, aryl hydrocarbon receptor; BA, bile acid; CCT, computerized cognitive training; CSF, cerebrospinal fluid; FDG, fluorodeoxyglucose; fNIRS, functional near-infrared spectroscopy; HIF-1α, hypoxia-inducible factor 1 alpha; LPS, lipopolysaccharide; MCI, mild cognitive impairment; MMSE, Mini-Mental State Examination; MPO, myeloperoxidase; pAD, prodromal Alzheimer’s disease; PET, positron emission tomography; PXR, pregnane X receptor; SCFA, short-chain fatty acid; SCD, subjective cognitive decline; sIgA, secretory immunoglobulin A; TMAO, trimethylamine N-oxide.

**Figure 8:**
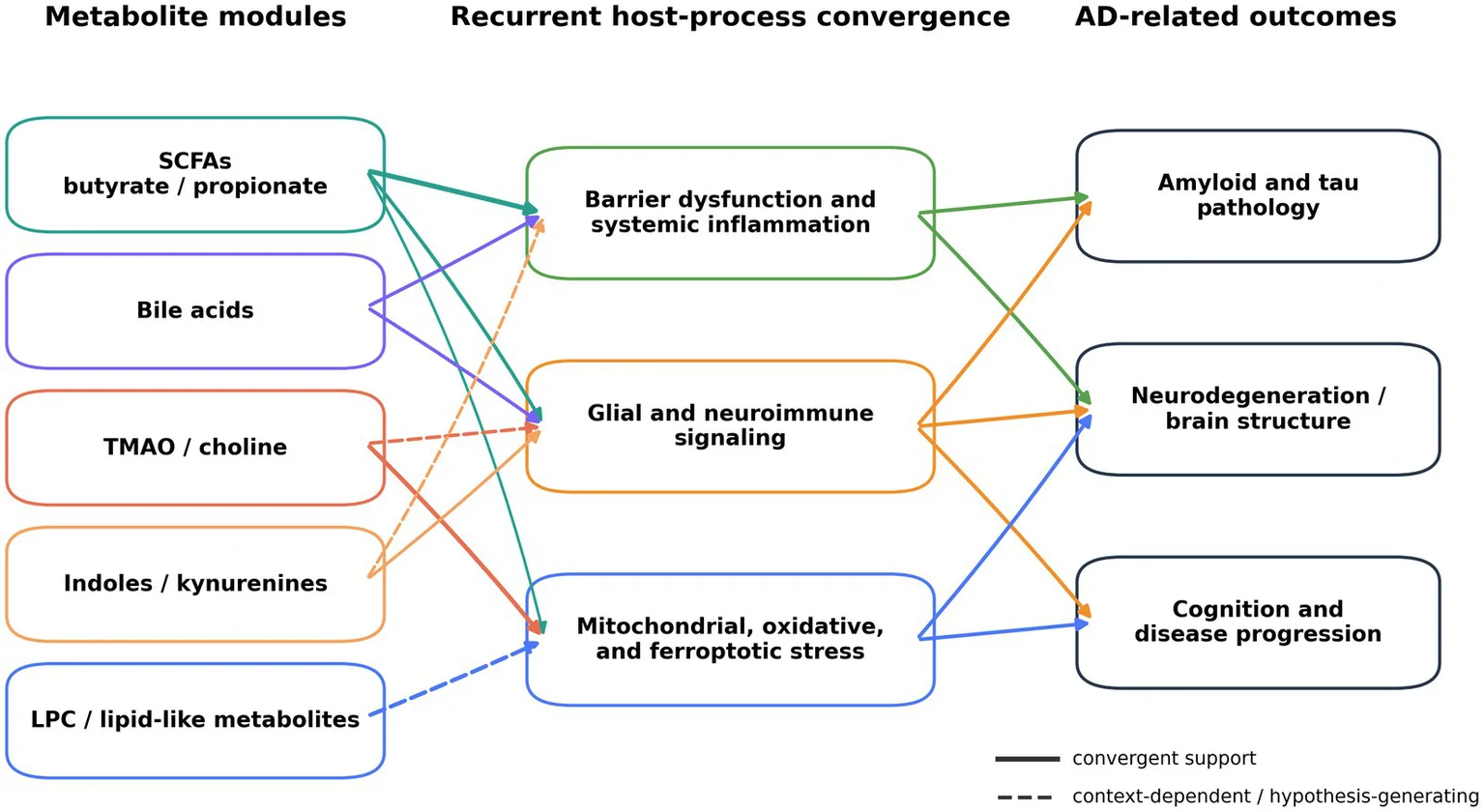
Evidence-weighted metabolite-host-brain knowledge graph. The module-level display links five metabolite modules to recurrent convergence on barrier dysfunction and systemic inflammation, glial and neuroimmune signaling, and mitochondrial, oxidative, and ferroptotic stress, followed by three AD-related outcome domains. Solid lines indicate convergent support; dashed lines indicate context-dependent or hypothesis-generating relationships. The complete 105-edge list and qualifiers remain in Supplementary Table S8. The graph is an evidence map rather than a causal model.

**Figure 9:**
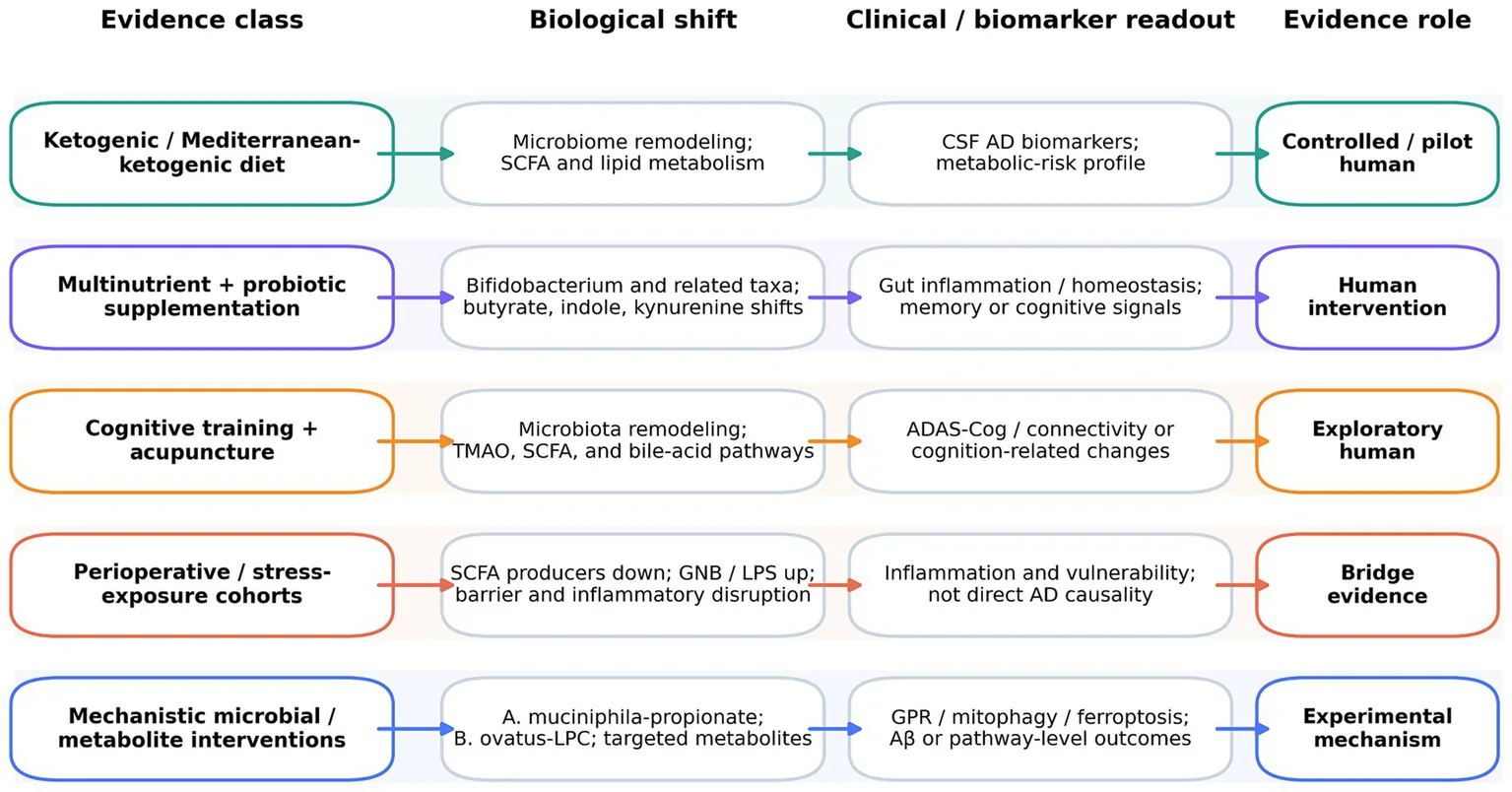
Intervention, bridge, and translational evidence map. Five evidence classes summarize principal biological shifts, clinical or biomarker readouts, and evidence roles. Human interventions inform translation; perioperative or stress-exposure cohorts provide bridge evidence; animal and *in vitro* studies support mechanism rather than independent clinical efficacy. Detailed study-level records remain in Supplementary Table S10. AD, Alzheimer’s disease; ADAS-Cog, Alzheimer’s Disease Assessment Scale-Cognitive Subscale; CSF, cerebrospinal fluid; GNB, Gram-negative bacteria; LPC, lysophosphatidylcholine; LPS, lipopolysaccharide; SCFA, short-chain fatty acid; TMAO, trimethylamine N-oxide.

**Figure 10:**
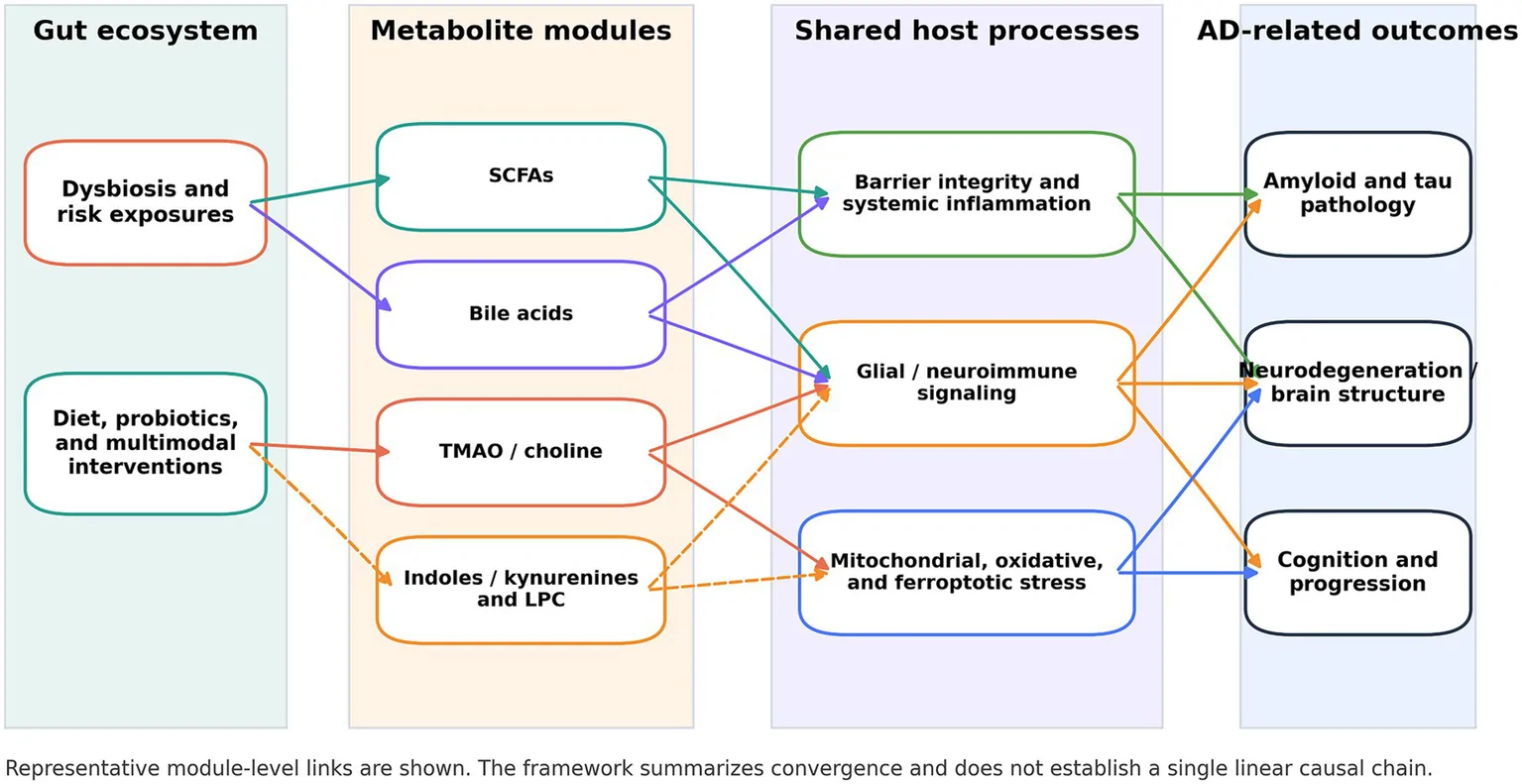
Integrated microbiota-metabolite-brain framework. The four-stage schematic links gut ecosystem context, metabolite modules, shared host processes, and AD-related outcomes. Only representative module-level relationships are shown to emphasize convergence rather than a single linear causal chain. Solid arrows indicate evidence-supported links, whereas dashed arrows indicate context-dependent or hypothesis-generating links. The framework does not establish clinical causality. AD, Alzheimer’s disease; LPC, lysophosphatidylcholine; SCFA, short-chain fatty acid; TMAO, trimethylamine N-oxide.

### Search strategy and evidence assembly

The final database search and export process was completed during June 20–22, 2026; the export files are dated June 22, 2026. PubMed, Web of Science Core Collection, and EBSCO/Academic Search Premier were searched to provide biomedical and multidisciplinary coverage. Embase and Scopus were not included because institutional access was unavailable during the search period. Citation checking and targeted full-text retrieval were used as supplementary screening and evidence-verification procedures. Supplementary Table S1 provides the database-specific search syntax, original export filenames, and source counts. The exports contained 1,153 PubMed records, 797 Web of Science records, and 24 EBSCO records, totaling 1,974. Case-normalized exact digital object identifier (DOI) matching identified 611 duplicates, leaving 1,363 records for screening; export-batch and duplicate-audit details are reported in Supplementary Table S1.

Screening excluded 1,273 records at the title/abstract level. Ninety reports underwent full-text assessment; 30 were excluded, leaving 60 evidence records. Thirty-two were coded as direct, bridge, or intervention human evidence and 28 as mechanistic, translational, animal, cellular, resource, or background-mechanism evidence. Supplementary Tables S1–S3 document the database counts, PRISMA arithmetic, and category-level full-text exclusion reasons.

### Eligibility criteria

Human core studies enrolled adults across the cognitive-aging continuum and directly measured microbiota-related metabolites or pathways in relation to cognition, AD pathology, neuroimaging, inflammation, barrier markers, or intervention response. Human bridge evidence was a human study outside the direct AD-core definition that measured a relevant metabolite or pathway against cognition, brain structure/function, barrier or inflammatory outcomes, or intervention response in a cognitively relevant population. Bridge evidence was not treated as direct confirmation of an AD-specific association. Broader stress-physiology and bidirectional immune–microbiome studies were included only when they met these metabolite-centered criteria or the prespecified bridge definition.

Animal, cellular, organoid, nematode, computational, and resource studies were eligible only when they experimentally tested or annotated a pathway relevant to AD or cognitive impairment. They supported biological plausibility and graph annotation but were not converted into human clinical claims.

Eligibility was restricted to studies that reported a measured or explicitly annotated microbiota-related metabolite or pathway in relation to an AD, MCI, or cognitive-aging outcome. Studies reporting taxonomic composition alone, metabolomics findings without a microbiota-related link, or no relevant cognitive or AD-related outcome were excluded. Reviews, non-research records, reports lacking extractable data on the population, metabolite or pathway, outcome, or effect direction, and overlapping publications without a unique contribution were also excluded.

### Data extraction and classification

Data were extracted for citation, study design, cohort and population characteristics, sample size, disease stage, biological matrix, analytical platform, metabolite module, direction of effect, outcome layer, evidence role, and risk-related considerations. Studies containing both human and experimental components were coded separately by component to avoid interpreting experimental findings as human causal evidence (Supplementary Tables S4, S5).

Outcome layers included cognition; amyloid, tau, and neurodegeneration (A/T/N) biomarkers; neuroimaging; inflammation and gut-barrier markers; mitochondrial or autophagy pathways; ferroptosis or oxidative stress; microglia; and synaptic or neuronal integrity. Cohort and study-network flags prevented multiple publications from being interpreted as independent replications.

### Synthesis approach

A broad meta-analysis was not undertaken because matrices, analytical platforms, metabolite annotation, disease stages, outcomes, covariate adjustment, and effect metrics were not sufficiently comparable. Unresolved or tentative metabolite annotation and the compositional nature of microbiome abundance data were also treated as comparability constraints (Da Silva et al., 2015; Forry et al., 2026). Quantitative summaries were used only within modules meeting comparability criteria. Otherwise, studies were grouped by module, stage, matrix, and outcome and synthesized using a structured direction-of-effect approach consistent with SWiM reporting principles (Campbell et al., 2020); module decisions are recorded in Supplementary Table S6.

Findings were coded as increased, decreased, mixed, pathway-reorganized, intervention-responsive, or insufficient, using adjusted primary analyses where available. Conflicting findings remained mixed rather than being resolved by vote counting. Direction scores were visual aids, not pooled estimates.

For Alzheimer’s Disease Neuroimaging Initiative (ADNI) bile-acid reports, cohorts, exposures, biomarkers, and outcome layers were mapped separately. Analyses based on overlapping participants and the same underlying relationship were treated as complementary analyses rather than additive samples or independent replications (Supplementary Table S7).

### Knowledge graph construction and evidence weighting

Knowledge-graph edges were extracted as citation-linked source–relationship–target statements connecting microbiota or interventions, metabolites, host pathways, AD biomarkers, neuroimaging, and cognition. An edge was retained only when its source and relationship could be traced to a specific report and the relationship was directly reported, experimentally manipulated, or explicitly designated as a cross-module synthesis link.

Edge prominence was represented using a prespecified three-level ordinal weighting scheme: weight 3 for human intervention evidence or relationships supported by replicated or longitudinal human studies; weight 2 for single human observational studies or directly manipulated mechanistic evidence; and weight 1 for bridge, resource, or review-based support. Evidence tier, independence across study families, directional conflict, cohort overlap, and major limitations were retained as separate attributes rather than combined into an overall score. No formal centrality ranking was performed. Instead, retained edges were compared at the module level according to evidence tier, study-family independence, breadth of AD-related outcome coverage, and context-dependent conflict or limited human validation.

The workflow evaluated 121 candidate relationships and retained 105 citation-level edges after de-duplication and traceability screening; 16 redundant or insufficiently traceable candidates were excluded. Supplementary Table S8 provides the retained edges and their ordinal evidence attributes. The weighting scheme was used to indicate relative evidence prominence and support module-level interpretation, not to estimate causal effects, clinical certainty, or treatment priority.

### Risk-of-bias and qualitative evidence assessment

Risk of bias was assessed by design: the Cochrane Risk of Bias 2 tool (RoB 2) for randomized trials (Sterne et al., 2019), Risk Of Bias In Non-randomized Studies of Interventions (ROBINS-I) for non-randomized interventions (Sterne et al., 2016), the relevant Joanna Briggs Institute (JBI) checklist for cross-sectional, case–control, or cohort studies (Aromataris et al., 2024), the Prediction model Risk Of Bias ASsessment Tool (PROBAST) for prediction models (Wolff et al., 2019), and the SYRCLE risk-of-bias tool for animal experiments (Hooijmans et al., 2014). Mixed human-animal studies were evaluated by component; *in vitro*, computational, and resource components used a domain-based mechanistic appraisal addressing model relevance, experimental manipulation, dose plausibility, replication, and human support. Supplementary Table S9 identifies the primary tool for each study or component.

Module-level evidence descriptors were limited, moderate, or stronger. These are review-specific qualitative categories based on human-evidence directness, consistency across independent study families, longitudinal or intervention support, mechanistic plausibility, and major limitations. They are not GRADE ratings and do not represent pooled effect sizes or clinical recommendations (Guyatt et al., 2008). Risk-of-bias assessments were completed for 36 study-level or resource records; methodological guidance and general background citations were outside that appraisal.

## Results

### Search and evidence assembly

The evidence assembly workflow identified 1,974 records from database searches before deduplication. After removing 611 duplicate records, 1,363 unique records were screened and 1,273 records were excluded at title/abstract triage. Ninety reports underwent full-text assessment; 30 were excluded, leaving 60 evidence records for tiered synthesis. Of these, 32 supported direct, bridge, or intervention human evidence and 28 supported mechanistic, translational, animal, cellular, resource, or background-mechanism interpretation.

Recent reports published from 2024 to 2026 contributed particularly to bile-acid biomarker analyses, SCFA pathway studies, microbiota-metabolite-brain multiomics, and intervention studies targeting the microbiota-gut-brain axis. A corrigendum related to the microbiome-derived indole-3-lactic acid/AhR article was retained as a bibliographic note and was not counted as a separate original study.

### Characteristics of the included evidence

The included evidence spans cognitively unimpaired adults, amyloid-positive preclinical AD, subjective cognitive decline, amnestic MCI, MCI due to AD, mild AD, AD dementia, and AD-risk intervention populations. Sample matrices included feces, serum, plasma, cerebrospinal fluid, and multi-biofluid metabolomics. Analytical platforms included targeted metabolomics, untargeted LC–MS/GC–MS/NMR metabolomics, fecal volatile organic compound profiling, 16S rRNA sequencing, shotgun metagenomics, metagenomic pathway analysis, proteomics, lipidomics, neuroimaging, CSF biomarker panels, PET biomarker data, and inflammatory or gut-barrier marker assays.

Most early human studies focused on single pathways, such as CSF TMAO or serum bile acids. More recent studies increasingly integrate microbiota, metabolites, brain imaging, CSF or PET biomarkers, inflammation, and cognition, enabling multi-layer synthesis across the cognitive-aging continuum. Intervention evidence includes ketogenic or Mediterranean ketogenic diet, multinutrient supplementation, probiotic supplementation, computerized cognitive training, microbial supplementation, and metabolite supplementation in translational models.

A compact overview of the included evidence base and analytic use is provided in Table 1.

**Table 1:** Summary of the included evidence base and analytic use.

Evidence domain	Coverage	Core measurements	Role in synthesis
Total evidence pool	60 records: 32 direct, bridge, or intervention human evidence records and 28 mechanistic, translational, animal, cellular, resource, or background-mechanism records	Human cognitive-aging continuum and translational models; fecal, blood, CSF, imaging, and multiomics data	Tiered synthesis and PRISMA-linked audit trail
SCFAs	Human and mechanistic evidence across preclinical AD, MCI, and AD	Producer taxa and pathways; fecal and circulating SCFAs	Direction-of-effect and mechanistic synthesis
Bile acids	ADNI-linked and external human analyses	Serum bile acids; CSF, PET/MRI, and cognitive outcomes	Biomarker synthesis with cohort-overlap controls
TMAO/choline	Human biomarker, intervention, and mechanistic evidence	CSF/plasma TMAO and choline-related pathways	Context-dependent biomarker synthesis
Tryptophan/indole/kynurenine	Emerging human and mechanistic evidence	Kynurenine/tryptophan balance and indole pathways	Hypothesis-generating immune-neurochemical module
Multiomics and barrier-inflammation	Human multiomics and bridge evidence	Metabolites, cytokines, barrier markers, imaging, and cognition	Cross-domain convergence
Intervention and translational evidence	Human interventions and mechanistic models	Dietary, probiotic, multinutrient, and pathway-directed interventions	Modifiability and trial prioritization
Evidence-weighted knowledge graph	121 candidate relationships; 105 retained edges	Metabolites, host pathways, AD biomarkers, imaging, and cognition	Ordinal evidence map

AD, Alzheimer’s disease; CSF, cerebrospinal fluid; MCI, mild cognitive impairment; MRI, magnetic resonance imaging; PET, positron emission tomography; SCFA, short-chain fatty acid; TMAO, trimethylamine N-oxide. Detailed study characteristics, evidence coding, risk-of-bias records, intervention fields, and knowledge-graph edges are provided in Supplementary Tables S4–S10.

### Evidence landscape across the cognitive-aging continuum

The evidence landscape has shifted from descriptive case–control comparisons toward integrated multiomics and interventional designs. SCFAs, bile acids, TMAO/choline-related metabolites, and tryptophan/indole/kynurenine metabolites remain the most frequently studied modules. Evidence density is greatest in MCI and AD dementia, while amyloid-stratified cohorts and functional metagenomic studies are extending the field into preclinical AD.

Across modules, direct comparison was limited by differences in disease stage, biological matrix, analytical platform, metabolite annotation, covariate adjustment, and cohort overlap. Direction-of-effect synthesis identified altered SCFA-related function and exposure, disrupted bile-acid profiles, context-dependent TMAO/choline findings, and less extensively studied tryptophan-related pathways. Associations across these modules involved barrier-related, inflammatory or glial, and mitochondrial outcome domains, but the available evidence did not establish a single causal chain linking these domains.

Table 2 summarizes the core metabolite modules, review-specific evidence profiles, and interpretive cautions used for this tiered synthesis.

**Table 2:** Core metabolite modules: review-specific evidence profile and interpretation.

Metabolite module	Main human evidence	Mechanistic support	Review-specific evidence profile and main caution
SCFAs and SCFA-producing capacity	Altered producer taxa or pathways and fecal/circulating SCFAs across preclinical AD, MCI, and AD.	Barrier, immune, microglial, mitochondrial, and GPR41/GPR43-related pathways.	Moderate; measurement layers are non-equivalent and do not support a uniform concentration direction.
Bile-acid profiles	Associations with cognition, CSF biomarkers, PET/MRI, and longitudinal pathology in ADNI-linked and external cohorts.	Host-microbial transformation and FXR/TGR5, lipid, inflammatory, and barrier signaling.	Stronger within this review; cohort overlap and heterogeneous exposures limit independent replication and causal interpretation.
TMAO and choline-related metabolites	CSF associations contrast with null or renal-function-dependent plasma findings.	Oxidative-stress, tau, GSK-3β, HIF-1α, and intervention-linked pathways.	Moderate and heterogeneous; matrix, kidney function, population, and covariate adjustment materially affect interpretation.
Tryptophan, indole, and kynurenine pathways	Human intervention and metabolomic studies report altered kynurenine/tryptophan balance and indole-derived metabolites.	Indole metabolites and AhR/PXR-related amyloid, inflammatory, and cognitive pathways.	Limited to moderate; mechanistic plausibility exceeds direct human AD-biomarker validation.
Lipid-like metabolites and multiomics signatures	Multiomics studies identify cognition-associated lipid, inflammatory, sugar, oxylipin, and microbial-metabolite signals.	LPC-GPR119-ferroptosis and resource-supported candidate pathways.	Limited to moderate; assay harmonization and independent replication are needed.
Barrier-inflammation-brain signatures	Bridge studies link dysbiosis with LPS, barrier markers, cytokines, imaging, and cognition.	Gut-barrier dysfunction, systemic inflammation, BBB vulnerability, and microglial activation.	Limited to moderate; surgery, stress, and inflammatory comorbidity restrict direct AD interpretation.

AD, Alzheimer’s disease; AhR, aryl hydrocarbon receptor; BBB, blood–brain barrier; CSF, cerebrospinal fluid; FXR, farnesoid X receptor; GPR, G-protein-coupled receptor; GSK-3β, glycogen synthase kinase 3 beta; HIF-1α, hypoxia-inducible factor 1 alpha; LPC, lysophosphatidylcholine; LPS, lipopolysaccharide; MCI, mild cognitive impairment; MRI, magnetic resonance imaging; PET, positron emission tomography; PXR, pregnane X receptor; SCFA, short-chain fatty acid; TGR5, Takeda G-protein-coupled receptor 5; TMAO, trimethylamine N-oxide. Detailed direction-of-effect coding, operational evidence descriptors, cohort-overlap rules, and graph attributes are provided in Supplementary Tables S5–S8.

### Short-chain fatty acid module

Human SCFA evidence was heterogeneous because studies assessed non-equivalent layers: SCFA-producing taxa, microbial production capacity, fecal concentrations, circulating exposure, or intervention-related change. In preclinical AD, functional metagenomic analyses reported reorganization of SCFA-related pathways, whereas findings for individual fecal or circulating SCFAs varied across studies (Verhaar et al., 2022; Wu et al., 2021; Xi et al., 2021; Ubeda et al., 2023; Saji et al., 2020; González-Domínguez et al., 2021; Nagpal et al., 2019; Kuehn et al., 2025; Duan et al., 2021; Liu et al., 2022; Yamashiro et al., 2024; Dissanayaka et al., 2026; Zhao et al., 2025).

Experimental studies examined pathway-specific aspects of SCFA biology. Agathobacter rectalis and butyrate were associated with reduced microglial activation and neuroinflammation in experimental models, while *Akkermansia muciniphila* and propionic acid were linked to GPR41/GPR43-related mitochondrial pathways. These effects were endpoint specific, and several behavioral, mitochondrial, or synaptic measures remained unchanged in the latter study (Nagpal et al., 2019; Molinero et al., 2025; Wang et al., 2025; Lv et al., 2024).

### Bile-acid module

Bile-acid evidence was represented primarily by ADNI-derived and other large biomarker datasets, which provided multimodal CSF, imaging, and cognitive outcome layers (Blennow et al., 2010; Hampel et al., 2018; Weiner et al., 2017). Direct bile-acid studies associated serum profiles and ratios with cognition, AD diagnosis, CSF amyloid and tau biomarkers, amyloid and tau PET measures, MRI phenotypes, white-matter microstructure, and longitudinal pathology. More recent studies extended these findings to larger cohorts and longitudinal amyloid/tau outcomes (Nho et al., 2019; MahmoudianDehkordi et al., 2019; Nabizadeh et al., 2024; Mo and Peng, 2026; Fu et al., 2026).

A substantial proportion of the bile-acid evidence was derived from overlapping ADNI or related study families. These reports examined different outcome layers within partially overlapping cohorts rather than providing fully independent replications. Exposure definitions also varied across individual bile acids, secondary bile-acid patterns, and bile-acid ratios.

### TMAO, choline, indole, and kynurenine pathways

Human TMAO findings were matrix- and context-dependent. CSF TMAO was associated with MCI/AD status and tau or neurodegeneration markers in one biomarker study. In contrast, a large population-based analysis found no overall association between plasma TMAO and cognition, neuroimaging, or incident dementia. Adverse risk emerged mainly among participants with impaired renal function, whereas choline showed broader associations (Vogt et al., 2018; Yaqub et al., 2024; Connell et al., 2026).

Human studies of tryptophan, indole, and kynurenine pathways were less numerous. Probiotic and nutrition studies reported intervention-related changes in kynurenine/tryptophan metabolism or indole-derived metabolites, but direct associations with biomarker-defined AD outcomes were examined in only a small number of studies (Leblhuber et al., 2018; Baek et al., 2025).

Experimental TMAO studies examined HIF-1α- and GSK-3β-related signaling, oxidative stress, synaptic changes, and tau-related outcomes (Shi et al., 2025; Lv et al., 2026). Separate experimental studies of microbial tryptophan metabolites, including indole-3-propionic acid and indole-3-lactic acid, examined AhR/PXR-related immune and barrier signaling and reported changes in amyloid- and inflammation-related outcomes (Li et al., 2025; Kim et al., 2024).

### Multiomics, inflammation, and gut-barrier evidence

Multiomics studies reported associations across different combinations of microbial composition, fecal or circulating metabolites, inflammatory cytokines, gut-barrier markers, neuroimaging, and cognition. These components were not consistently measured within the same cohorts, and analytical pipelines differed across studies. In AD and MCI cohorts, metabolite perturbations co-occurred with inflammatory, gut-barrier, neuroimaging, and cognition-related changes. Selected mediation pathways linking microbiota, metabolites, neuroimaging, and cognition were examined in one study (Zhao et al., 2025), but no study tested the complete microbiota–metabolite–inflammation/barrier–brain pathway across all proposed layers (Hosseinkhani et al., 2025; Duan et al., 2021; Liu et al., 2022; Dissanayaka et al., 2026).

Prodromal and perioperative cohorts further reported reduced SCFA-producing bacteria, increased Gram-negative taxa, elevated lipopolysaccharide, changes in claudin or occludin, and systemic inflammatory alterations in relation to SCD, aMCI, or perioperative cognitive vulnerability. Most of these studies were cross-sectional, and the perioperative findings were obtained in settings involving surgery, medication exposure, or acute inflammatory stress (Zhao et al., 2025; Hosseinkhani et al., 2025; Duan et al., 2021; Liu et al., 2022; Dissanayaka et al., 2026). These cohorts were retained as context-specific bridge evidence within the synthesis.

### Intervention and translational evidence

Human intervention evidence included ketogenic or Mediterranean ketogenic diets, clinical multinutrient supplementation, probiotic supplementation in AD or MCI, *Lactobacillus delbrueckii* subsp. *lactis* CKDB001 supplementation, metformin treatment, acupuncture-associated multiomics interventions in aMCI, and computerized cognitive training. Multinutrient trials primarily evaluated cognitive or functional outcomes, whereas later work added gut-microbiome and intestinal-function measures (Scheltens et al., 2012; Soininen et al., 2017; Molinero et al., 2025). Across interventions that directly measured microbiome or metabolite endpoints, reported changes involved different combinations of microbial composition, fecal or circulating metabolites, inflammatory or metabolic biomarkers, neurofunctional measures, cognition, and AD-related biomarkers (Nagpal et al., 2019; Dilmore et al., 2023; Schweickart et al., 2024; Leblhuber et al., 2018; Baek et al., 2025; Yin et al., 2025; Zhang et al., 2025; Rosell-Díaz et al., 2024).

Human intervention studies generally involved small or exploratory samples, short and variable follow-up, and heterogeneous intervention protocols and outcome definitions. Changes in microbial or metabolite measures were not consistently accompanied by changes in cognition or AD biomarkers, and most studies did not formally test whether metabolite shifts mediated the clinical, neurofunctional, or biomarker responses.

Experimental intervention studies examined model-specific pathways involving *Bacteroides ovatus*–LPC–GPR119–ferroptosis, Agathobacter rectalis–butyrate–Akt/NF-κB–microglial signaling, *Akkermansia muciniphila*–propionate–GPR41/GPR43–mitochondrial homeostasis, TMAO-related HIF-1α/GSK-3β, oxidative-stress and tau pathways, and microbial indole signaling. Reported endpoints included amyloid- and tau-related changes, ferroptosis, mitochondrial or mitophagy measures, microglial and inflammatory responses, and cognitive or other neurodegeneration-related phenotypes in animal, cellular, nematode, or organoid models (Wang et al., 2025; Zha et al., 2025; Lv et al., 2024; Zhang et al., 2024; Li et al., 2025; Shi et al., 2025; Lv et al., 2026; Kim et al., 2024).

Table 3 summarizes the intervention, bridge, and mechanistic evidence categories; detailed study-level fields are provided in Supplementary Table S10.

**Table 3:** Summary of intervention, bridge, and translational evidence.

Evidence class or model	Evidence tier	Main pathways and outcomes	Interpretation
Ketogenic and Mediterranean-ketogenic diets	Human pilot or AD-risk intervention evidence	SCFA/microbiome and serum/CSF metabolomic changes; AD biomarkers and metabolic or cognitive profiles	Supports dietary modifiability; small samples, adherence, and matrix heterogeneity limit causal inference.
Multinutrient and probiotic supplementation	Human exploratory or randomized intervention evidence, with some ex vivo components	SCFA-related and kynurenine/indole pathways; cognition and gut, barrier, or inflammatory measures	Provides controlled human signals; follow-up duration and biomarker depth remain limited.
Cognitive training and acupuncture-associated multiomics	Human intervention or bridge evidence	TMAO or serum-metabolite networks; cognition and neurofunctional measures	Hypothesis-generating; mediation, specificity, and replication remain unproven.
Perioperative and prodromal bridge cohorts	Observational bridge evidence	SCFA-producing bacteria, LPS, barrier and inflammatory markers; cognitive vulnerability	Supports a barrier-inflammation-brain link, but surgery and stress are major confounders.
Mechanistic microbial or metabolite interventions	Animal, cellular, nematode, organoid, or resource evidence	SCFA, LPC, TMAO, and indole pathways; amyloid, tau, ferroptosis, microglia, and mitochondrial outcomes	Strengthens biological plausibility; not evidence of human efficacy.

AD, Alzheimer’s disease; CSF, cerebrospinal fluid; LPC, lysophosphatidylcholine; LPS, lipopolysaccharide; SCFA, short-chain fatty acid; TMAO, trimethylamine N-oxide. Study-level intervention designs, metabolite and outcome fields, and limitations are provided in Supplementary Table S10.

### Evidence-weighted knowledge graph

The evidence-weighted knowledge graph retained 105 of 121 candidate citation-level relationships after de-duplication and traceability screening. The retained network connected SCFA-related pathways, bile-acid metabolism, TMAO/choline metabolism, indole/tryptophan/kynurenine metabolism, and lipid-like metabolites with host processes, AD biomarkers, neuroimaging, and cognition. Resource-based metabolite–receptor mappings were used only for annotation and hypothesis generation, and no formal centrality ranking was performed (Qiu et al., 2024).

Examination of the retained edges across evidence tier, study-family independence, outcome coverage, and conflict qualifiers revealed three distinct patterns. Bile-acid ratios and secondary bile-acid profiles, together with SCFA-producing capacity and related functional pathways, formed branches with relatively broad human outcome coverage but continuing needs for independent replication because of cohort overlap or non-equivalent measurement layers. The TMAO/choline branch was characterized by context-dependent conflict, including positive CSF associations, null overall findings in population-based plasma studies, and renal-function-dependent effects. Indole/kynurenine- and lysophosphatidylcholine-related branches contained comparatively extensive mechanistic evidence but limited direct validation in biomarker-defined human AD cohorts.

The graph therefore did not identify a single topological hub metabolite. Instead, it distinguished branches suited to independent human replication, relationships requiring context-specific conflict resolution, and experimentally supported pathways requiring targeted human validation. Detailed citation-level edges and qualifiers are provided in Supplementary Table S8.

### Risk of bias and qualitative evidence profile

Review-specific qualitative evidence was comparatively stronger for recurrent bile-acid associations with AD biomarkers and for SCFA-related pathways, although bile-acid evidence was concentrated in ADNI and related collaborative study networks. TMAO/choline evidence was moderate but heterogeneous across matrix, renal function, population, and confounder adjustment. Direct SCFA concentration and intervention findings were moderate and matrix dependent. Direct human AD-biomarker evidence for indole/tryptophan/kynurenine pathways and lipid-like microbial metabolites was more limited than the available mechanistic evidence (Supplementary Figure S1).

Cross-sectional designs, small samples, non-equivalent matrices and platforms, inconsistent metabolite nomenclature, variable confounder adjustment, tentative feature annotation, microbiome compositionality, and concentration within a limited number of cohorts reduced cross-study comparability. No broad pooled estimate was generated, and findings were summarized at the module level (Supplementary Figure S2).

## Discussion

### Principal findings

This review shows that microbiota-derived metabolite evidence in AD has moved beyond isolated case–control observations, although evidence maturity differs substantially across modules. Across the cognitive-aging continuum, recurrent modules include SCFA production and exposure, bile-acid host-microbial co-metabolism, TMAO/choline metabolism, tryptophan/indole/kynurenine pathways, and emerging lipid-like microbial metabolites. These modules intersect with barrier integrity, inflammatory and glial signaling, mitochondrial stress, ferroptosis, amyloid/tau biology, neuroimaging, and cognition without establishing a single causal chain.

Two conclusions follow. First, bile-acid profiles and SCFA-related functional capacity currently have the broadest combination of human and translational support, although bile-acid findings are constrained by cohort overlap and SCFA findings depend strongly on the measurement layer. TMAO/choline associations remain context dependent, whereas tryptophan-related and lipid-like pathways are supported mainly by mechanistic or hypothesis-generating evidence. Second, differences in disease stage, biological matrix, analytical platform, covariate adjustment, and cohort overlap preclude treating all modules or evidence tiers as equivalent and do not support a single cross-module pooled estimate.

Human and mechanistic studies addressed different questions and were interpreted separately. Human observational studies informed associations with cognition, AD biomarkers, neuroimaging, and clinical stage, whereas human intervention studies provided evidence on modifiability. Animal, cellular, organoid, nematode, and in silico studies were used to examine pathway plausibility and generate mechanistic hypotheses. Agreement between human and experimental findings increased the biological coherence of a proposed pathway but did not establish mediation, clinical efficacy, causality, or independent human validation.

### Cross-study heterogeneity and unresolved questions

Five recurrent sources of heterogeneity likely contributed to cross-study differences: disease stage, biological matrix, analytical platform and metabolite annotation, statistical adjustment, and cohort overlap. Fecal signals cannot be assumed to represent circulating or central exposure, and pathways inferred from microbial genes are not equivalent to directly measured metabolite concentrations. Diet, medication use, renal function, cardiometabolic status, and comorbidities further influenced the interpretation of reported associations.

These sources of heterogeneity indicate that apparently discordant findings do not necessarily represent direct contradictions. Key unresolved questions include whether SCFA patterns primarily reflect microbial production or host absorption and utilization; whether bile-acid signatures can be replicated outside overlapping study families; whether TMAO provides independent biomarker or prognostic information after accounting for renal function and cardiometabolic status; and whether tryptophan-related pathways with extensive mechanistic support can be validated in biomarker-defined human AD cohorts.

### SCFAs as a modifiable but context-dependent module

SCFA findings must be interpreted by measurement layer. Producer abundance, microbial pathway capacity, fecal concentration, circulating exposure, and intervention response address different biological questions. The human literature does not show a uniform change in one fecal or circulating SCFA, and apparent disagreement may reflect diet, gut transit, cross-feeding, absorption, host utilization, or excretion rather than direct contradiction. Experimental SCFA studies provide plausible links to inflammatory, microglial, and mitochondrial pathways, but whether these mechanisms explain SCFA-related findings in human AD remains uncertain. Effects observed after direct microbial or metabolite administration in experimental models cannot be assumed to represent naturally occurring fecal or circulating SCFA variation in humans.

### Bile acids and AD pathology

Bile acids are among the more extensively characterized human biomarker modules in this literature. Serum bile-acid profiles and ratios have been associated with cognition, AD diagnosis, CSF amyloid and tau biomarkers, amyloid and tau PET, FDG-PET, MRI phenotypes, white-matter microstructure, and longitudinal pathology. However, much of this outcome breadth derives from layered analyses of ADNI or ADNI-related resources rather than independent replication across populations.

These findings should therefore be interpreted as complementary human biomarker layers rather than independent replications of a single exposure–outcome association. The available evidence remains primarily observational and does not identify a single causal bile-acid species or establish that bile-acid alterations mediate AD progression. Bile-acid ratios and secondary bile-acid patterns may reflect altered gut–liver–brain regulation, but this interpretation requires independent replication and stage-specific longitudinal testing.

FXR/TGR5 signaling, lipid metabolism, inflammation, and blood–brain barrier vulnerability provide candidate mechanistic explanations for these associations. At present, however, these pathways remain hypothesis-generating and have not been demonstrated as mediators of the human biomarker findings.

### TMAO and tryptophan-related immune-neurochemical pathways

TMAO does not provide a uniformly adverse human signal across biological matrices or populations. CSF findings support associations with tau and neurodegeneration, whereas population-based plasma data include null overall results and effect modification by renal function. Choline and related precursors may also show associations distinct from TMAO itself. Kidney function, diet, cardiovascular disease, medication use, and analytical matrix are therefore central to the interpretation of the human findings. Experimental TMAO studies involving HIF-1α/GSK-3β signaling, oxidative stress, synaptic changes, and tau-related pathways provide mechanistic context but do not establish that these mechanisms mediate TMAO-related outcomes in human AD.

Human evidence for tryptophan, indole, and kynurenine pathways remains limited. Probiotic and nutritional studies have reported changes in kynurenine/tryptophan metabolism or indole-derived metabolites, but direct associations with biomarker-defined AD outcomes have been examined in only a few studies. Experimental evidence is more extensive: microbial tryptophan metabolites have been linked to AhR/PXR-related immune and barrier signaling and to amyloid- or inflammation-related phenotypes in model systems. This module should therefore be regarded primarily as an area of mechanistic support and hypothesis generation, with the experimental pathways used to prioritize targeted measurements rather than interpreted as established human clinical mechanisms.

### Biological interpretation and graph-derived hypotheses

The main biological insight from the evidence-weighted knowledge graph was not the identification of a single metabolite hub, but the observation that substantial diversity at the metabolite level narrowed to a smaller set of recurrent host responses. Five upstream metabolite modules repeatedly converged on two broad process clusters: barrier dysfunction, systemic inflammation, and glial or neuroimmune signaling; and mitochondrial, oxidative, and ferroptotic stress. This pattern is difficult to recognize from a study-by-study narrative because the relevant evidence is distributed across different biological matrices, disease stages, outcome layers, and experimental systems.

Convergence does not imply that these relationships form a single causal pathway. Rather, it suggests that distinct metabolic disturbances may intersect with partially shared host vulnerabilities. Bile-acid and SCFA-related branches were anchored by comparatively broad human outcome coverage but remained limited by cohort overlap and differences between functional capacity, fecal concentration, and circulating exposure. TMAO/choline occupied a different position because its apparent associations varied with biological matrix, renal function, and population context. Indole/kynurenine- and lysophosphatidylcholine-related branches showed the opposite imbalance: detailed experimental pathways were available, but direct human AD validation remained sparse.

The graph supports several testable propositions. Microbial SCFA-producing capacity may be more reproducibly associated with barrier and inflammatory markers than any single fecal or circulating SCFA concentration. Bile-acid ratios and secondary bile-acid patterns may replicate more consistently across cohorts and disease stages than individual bile-acid species. Associations between TMAO and AD-related outcomes may change substantially after accounting for renal function and biological matrix. Finally, indole- and lysophosphatidylcholine-related pathways would be more meaningfully evaluated when the metabolite, its proposed receptor or host-response intermediate, and AD biomarker outcomes are measured in the same participants. These propositions are graph-derived priorities for testing rather than established causal or clinical conclusions.

### Intervention evidence and current translational limits

Current human intervention studies indicate that microbial and metabolite features can change following dietary, nutritional, microbiome-targeted, or multidomain interventions. However, heterogeneous intervention protocols, small or exploratory samples, short and variable follow-up, inconsistent cognitive and biomarker endpoints, and limited mediation analyses prevent attribution of the observed responses to a specific metabolite pathway. Experimental intervention studies identify manipulable candidate mechanisms under controlled conditions, but they do not establish clinical efficacy or demonstrate that the same pathways mediate cognitive or biomarker responses in humans.

## Strengths and limitations

This review prioritizes human evidence while using experimental studies to examine biological plausibility rather than as substitutes for clinical evidence. It avoids broad pooling across non-comparable metabolomics studies, treats overlap among ADNI-derived analyses as an explicit interpretive issue, and maintains a traceable record of study selection, risk-of-bias assessment, and knowledge-graph evidence.

Several limitations should be considered. The protocol was not prospectively registered, and Embase and Scopus were not searched because institutional access was unavailable; relevant studies indexed exclusively in these databases may therefore have been missed. The evidence base was dominated by observational or cross-sectional designs, small samples, heterogeneous analytical platforms and metabolite nomenclature, and inconsistent adjustment for major confounders. The metabolite-centered eligibility criteria improved the focus of the synthesis but may have excluded exploratory microbiome or metabolomics studies without an explicit microbiota–metabolite link, a relevant cognitive or AD-related outcome, or sufficiently extractable evidence. Geographic representation was also uneven, with the detailed human reports concentrated in East Asia, North America, and Europe. Finally, stool, blood, urine, and CSF measurements are not interchangeable, and mixed disease-stage samples may obscure stage-specific associations. Experimental and resource-based evidence was therefore used for mechanistic interpretation and graph annotation, not as human effect estimates; overlapping ADNI bile-acid reports were treated as complementary outcome layers rather than independent replications.

### Implications for future research

Near-term research should focus on prospective, stage-stratified human cohorts that measure microbial function, fecal and circulating metabolites, AD biomarkers, cognition, and major confounders in the same participants. Intervention studies should move beyond reporting changes in microbial composition or metabolite abundance and formally test whether metabolite shifts mediate biomarker or cognitive responses. Independent validation should then target the principal graph-derived gaps using prespecified biological matrices, appropriate covariate adjustment, and joint measurement of metabolites, candidate host-response pathways, and AD-related outcomes. Harmonized reporting and transparent handling of overlapping cohorts will be essential for subsequent quantitative synthesis.

## Conclusion

Across the cognitive-aging continuum, microbiota-derived metabolite signatures provide a functional layer associated with AD-related biology, but the evidence does not support a single causal metabolite pathway. Bile acids, distinct SCFA measurement layers, context-dependent TMAO/choline-related signals, tryptophan/indole/kynurenine metabolites, and lipid-like microbial metabolites are associated with barrier integrity, inflammation, mitochondrial homeostasis, ferroptosis, microglial activation, amyloid, tau, and cognition. The next step is prospective and interventional testing of whether modifying these modules changes AD biomarkers or cognitive trajectories.

## Data Availability

The original contributions presented in the study are included in the article/Supplementary material, further inquiries can be directed to the corresponding author/s.

## Supplementary material

Supplementary Figure S1 Qualitative structured evidence profile by metabolite module. Human-evidence directness, cross-study consistency, and mechanistic plausibility are displayed as limited, moderate, or stronger using the operational definitions in Supplementary Table S6. These are review-specific, non-numerical, non-GRADE descriptors

Supplementary Figure S2 Risk-of-bias and evidence-role summary for 36 appraised study-level or resource records. Panel **A** displays the distribution of overall risk-of-bias judgments recorded in Supplementary Table S9; Panel **B** displays the evidence roles retained for interpretation
