# Supplementary material for: Microbiota-derived metabolite signatures in Alzheimer’s disease across the cognitive aging continuum: a human-focused systematic review and evidence-weighted knowledge graph
Source: Front Microbiol. 2026 Sep 28;17:1925387. doi: 10.3389/fmicb.2026.1925387 (PMC13656208; doi:10.3389/fmicb.2026.1925387)
Supplement: Supplementary file 1 [file Supplementary_file_1.docx]

# 1 **Supplementary Material**

## 1.1 **Supplementary files in first-citation order**

Supplementary Tables S1–S12 and Supplementary Figures S1–S2 are provided as supplementary material and arranged in the order of first citation in the main manuscript.

Supplementary Table S1. Database search syntax, source counts, and de-duplication verification. File: Supplementary_Table_S1.xlsx.

Supplementary Table S2. PRISMA identification and screening count reconciliation. File: Supplementary_Table_S2.xlsx.

Supplementary Table S3. Category-level full-text exclusion reasons; the eight categories sum to 30. File: Supplementary_Table_S3.xlsx.

Supplementary Table S4. Characteristics, study settings, and geographic regions for 34 reports emphasized in the synthesis. File: Supplementary_Table_S4.xlsx.

Supplementary Table S5. Direction-of-effect matrix by metabolite module, sample type, disease stage, and outcome layer. File: Supplementary_Table_S5.xlsx.

Supplementary Table S6. Quantitative-summary feasibility, synthesis decisions, and operational evidence descriptors. File: Supplementary_Table_S6.xlsx.

Supplementary Table S7. ADNI cohort-overlap handling for bile-acid and biomarker analyses. File: Supplementary_Table_S7.xlsx.

Supplementary Table S8. Retained knowledge-graph edges, candidate accounting, and evidence attributes for 105 retained edges. File: Supplementary_Table_S8.xlsx.

Supplementary Table S9. Design-specific risk-of-bias assessment for 36 appraised records. File: Supplementary_Table_S9.xlsx.

Supplementary Table S10. Intervention, bridge, and translational evidence map. File: Supplementary_Table_S10.xlsx.

Supplementary Figure S1. Qualitative evidence profile by metabolite module using review-specific, non-GRADE descriptors. File: Supplementary_Figure_S1.tif.

Supplementary Figure S2. Risk-of-bias and evidence-role summary for 36 appraised records. File: Supplementary_Figure_S2.tif.

Supplementary Table S11. Figure/table data-source and synchronization dictionary. File: Supplementary_Table_S11.xlsx.

Supplementary Table S12. Bibliographic status of references published in 2025–2026. File: Supplementary_Table_S12.xlsx.
